# Academic Burnout and Parent–Child Discrepancies in Educational Expectations Among Chinese Adolescents: A Moderated Mediation Model

**DOI:** 10.3390/bs15070876

**Published:** 2025-06-27

**Authors:** Xuening Fan, Anna Na Na Hui

**Affiliations:** Department of Social and Behavioral Sciences, City University of Hong Kong, Hong Kong SAR, China

**Keywords:** academic stress, academic burnout, educational expectations, discrepancy, grit

## Abstract

Based on the Job Demands–Resources (JD-R) model, previous research has shown that burnout results from excessive demands combined with insufficient resources. This study evaluated the relationship between academic burnout and parent–child discrepancies in educational expectations, with the mediating role of academic stress and the moderating role of grit. A cross-sectional survey was conducted with 531 middle school students (Mage = 14.77, SD = 0.63; 47.08% female) in Zhengzhou, China, using self-report measures. The results showed that the expectation discrepancies were positively related to burnout, and academic stress partially mediated this association. In addition, grit moderated the effect of academic stress on burnout, indicating that grit could serve as a protective factor against academic burnout. These findings provide a better understanding of how expectation misalignment contributes to student burnout and highlight the protective role of grit. Suggestions for reducing academic stress and burnout among adolescents are discussed.

## 1. Introduction

Based on the results of the Programme for International Student Assessment (PISA) 2018, students from four Chinese provinces (i.e., Beijing, Shanghai, Jiangsu, and Zhejiang) aced the tests in reading, mathematics, and science, achieving scores that were significantly higher than their peers from 78 countries around the world (OECD, 2019). However, such outstanding performance has come at both physical and psychological cost. School-aged children in China constantly face high academic stress due to heavy school workloads, high expectations from parents and teachers, and overwhelming competitiveness among classmates (Sun et al., 2012). Academic stress has long been considered a significant factor not only hindering students’ academic success but also negatively affecting their physical and mental health (Shankar & Park, 2016; Liu & Lu, 2012). One strikingly negative syndrome attributed to prolonged academic stress is academic burnout, a psychological syndrome characterized by exhaustion, cynicism, and reduced efficacy in learning (Hao et al., 2022; Schaufeli et al., 2002).

Extensive research has been devoted to investigating factors that may lead to academic stress and symptoms of burnout in the unique cultural context of China, revealing family-related factors to be a major cause. Drawing on Identity Control Theory (ICT), which states that psychological distress can result when individuals perceive a mismatch between their internal identity standards and external feedback (Burke, 2007), such discrepancies during adolescence—particularly between students’ own educational expectations and what they perceive as their parents’ expectations—can disrupt identity verification and cause emotional strain.

To further understand the psychological mechanisms behind academic burnout, this study primarily adopts the Job Demands–Resources (JD-R) model, which indicates that burnout results from an imbalance between high demands and low resources (Demerouti et al., 2001). In an academic setting, excessive demands function as stressors, while internal resources such as grit (i.e., perseverance of effort and consistent interest in long-term goals) (Duckworth et al., 2007) may serve as protective factors. From this perspective, this study investigates whether the discrepancies between students’ educational expectations and their perceived parental expectations function as a form of academic demand that positively relates to stress and burnout, and whether grit could buffer some of the negative impacts of academic stress.

## 2. Literature Review

### 2.1. Discrepancies in Educational Expectations

According to Bronfenbrenner’s ecological systems theory, the development of children is constantly shaped by layers of the environments surrounding them (Bronfenbrenner, 1986). The most immediate levels of environment include settings such as family and school, which are also the ones that are most powerful in influencing children’s growth. The influence of family on students’ academic success has been examined from various perspectives, such as socioeconomic status, parental involvement, and parental expectations (Ma et al., 2018; Sengonul, 2022). In the Confucian cultural context, parents from many Asian countries place enormous emphasis on their children’s education and broadly hold rather high expectations (Tan & Yates, 2011). Confucian values stress the importance of obedience, pursuit of excellence and moral cultivation through learning, and these values may lead parents to set ambitious goals for their children’s academic outcomes and exert pressure and control over their educational choices (N. Zhu & Chang, 2019).

Educational expectations describe the realistic beliefs that parents, teachers, or students themselves hold concerning the highest educational achievement that a student could attain based on the capabilities and resources he or she possesses (Yamamoto & Holloway, 2010). Studies have demonstrated the crucial effects of parental educational expectations on children’s academic performance across a wide age range (Benner et al., 2021; Pinquart & Ebeling, 2020). However, while such expectations can serve as motivating factors, they may also negatively affect students’ emotional well-being. For example, high parental expectations have been linked with increased levels of depression among adolescents (Ma et al., 2018). These findings indicate that parental educational expectations may create stress and cause mental health problems for children, especially when the expectations are unrealistic or inconsistent with children’s own expectations (Rizwan et al., 2020).

Although much research has focused on parental educational expectations, a crucial yet understudied aspect is the level of agreement—or discrepancy—between students’ and parents’ expectations. Adolescence is a phase of identity development during which individuals go through a transitional change by actively and constantly shaping their self-concept (Cole et al., 2001). Based on Identity Control Theory (ICT), when feedback from others (i.e., perceptions) is not congruent with self-defining meanings (i.e., identity standards) in some situations, conflicting goals may arise and cause disruption in the process of identity verification, which may further lead to feelings of distress and depression (Burke, 2007). Accordingly, the disparities between adolescents’ own educational expectations (the identity standard) and their perception of external expectations from others in ICT may result in negative emotional effects. Evidence suggests that such discrepancies adversely affect students’ emotional well-being. For instance, upward-biased discrepancies, which refer to students perceiving that their parents’ expectations surpass their own, were associated with psychological stress and negative emotions (Cheng et al., 2022). When perceiving considerable discrepancies, the pressure of satisfying others’ expectations may arise, as L. F. Wang and Heppner (2002) indicated that the source of psychological distress was not parental expectations themselves but rather the attempt to live up to such expectations.

Another important methodological issue that needs to be addressed is how to measure such discrepancies between students and parents. Some studies have assessed expectation discrepancies by directly comparing students’ and parents’ reported expectations (e.g., Guo et al., 2022; Man et al., 2021). These studies use actual expectations—those reported by parents—rather than students’ perceptions of parental expectations. However, the current study relies on students’ perceived expectations, considering that ICT is centered on the subjects’ own perspectives and their perceptions of meaning rather than others’ points of view.

### 2.2. Academic Stress and Burnout in the Job Demands–Resources Model

Excessive academic stress has been recognized as a major contributor to students’ physical and mental health issues, including heightened risks of anxiety, depression, sleep disturbances, and obesity (Fu et al., 2022; X. Zhu et al., 2021). One of the negative consequences of prolonged stress is burnout. Burnout syndrome was initially brought up in the workplace environment, especially among professions that require interactions with people (Masłach, 1976). It refers to a psychological state of chronic stress, exhaustion, and reduced motivation that affects one’s performance and well-being. Previous research has shown that job burnout negatively affects employees’ performance, efficiency, and daily functioning (Bakker & Costa, 2014). Subsequently, the concept of burnout has been broadened to include all occupational groups, extending to students within academic settings (Schaufeli et al., 2002).

Academic burnout is defined through three dimensions: emotional exhaustion, cynicism, and inefficacy. Emotional exhaustion involves feeling emotionally and physically drained by stressful academic demands, while cynicism refers to students holding disengaged and distant attitudes toward schoolwork and possibly perceiving it as meaningless. Inefficacy refers to feeling incompetent in fulfilling certain academic tasks (Schaufeli & Salanova, 2007). Similar to findings regarding job burnout, studies have demonstrated that academic burnout was also associated with a series of external and internal issues, such as reduced academic performance, higher school dropout rates, and reduced psychological health (Bask & Salmela-Aro, 2013; Długosz & Liszka, 2021). These consequences have prompted extensive research on the contributing factors of academic burnout, among which high stress levels have been found to be a significant factor (Jung et al., 2015).

As one of the antecedents of burnout, psychological stress has been extensively discussed and explored in various models intended to clarify the mechanism that may lead to burnout (Lloyd et al., 2002). The Job Demands–Resources (JD-R) model is considered one of the leading models for predicting workplace burnout (Bakker & Demerouti, 2007). In particular, the model explains how demands and resources in the workplace interactively affect employees’ productivity and well-being through two distinct psychological paths. In the health impairment path, prolonged exposure to excessive demands in the workplace (e.g., tight deadlines, unfavorable working environment and workload) depletes energy levels, leading to disengagement and accumulated stress. In contrast, job resources (e.g., support, feedback, and good relationships) in the motivational path may lead to engagement and motivation. Sufficient job resources can help mitigate the adverse effects of job demands on individuals’ performance and well-being, but the overwhelming work demands along with a lack of coping resources will eventually result in burnout. Personal resources, such as resilience and self-efficacy, were subsequently added to the JD-R model as a reciprocal role with job resources and engagement (Bakker & Demerouti, 2014; Demerouti et al., 2001).

Similar to job burnout, the JD-R model was successfully transferred to school contexts. One of the first longitudinal studies applying the model to academic settings was conducted by Salmela-Aro and Upadyaya (2014), demonstrating that study demands were a positive predictor of burnout, while study resources positively predicted engagement after one year. The model was later tested in Chinese academic settings as Teuber et al. (2021) suggested that perceived school demands had a positive association with burnout and a negative association with engagement among Chinese high schoolers, which echoed the two paths in the original model. Academic demands in this case were correspondingly interpreted as job demands in the original JD-R model, a source of academic stress (Pascoe et al., 2020). Similar to job demands, academic demands comprise multiple aspects, including the perceived direct workload, curriculum difficulty, and exam pressure (Giota & Gustafsson, 2017). Additionally, the expectation discrepancies can be regarded as an indirect academic demand since they emphasize the demanding achievement gap that students must face between their expectations and perceived parental expectations. Therefore, as the JD-R model illustrates that excessive demands can cause stress and subsequent burnout, here, we reasonably speculated that the discrepancies, as one of the stressful academic demands perceived by students, would increase academic stress and subsequently trigger burnout.

Based on ICT, which states that discrepancies between self-defined meaning and perception may lead to stress, which in turn serves as the antecedent in the JD-R model inducing burnout, the present study intends to test the relationship between the expectation discrepancies and academic burnout through the mediator of academic stress, which has not yet been adequately explored.

### 2.3. The Moderating Role of Grit

The construct of grit is defined as the sustained perseverance and consistent interest in achieving long-term goals (Duckworth et al., 2007). While grit was initially viewed as a relatively unchanging personal trait over the lifespan (Duckworth & Quinn, 2009), studies have demonstrated its malleability with successful interventions (Park et al., 2020). These promising findings have provided more insights for future research, suggesting that grit can be cultivated and developed. The importance of grit is further emphasized uniquely in China since perseverance and diligence are highly valued characteristics in traditional Chinese culture (Harrell, 1985). It is believed that industriousness and determination lead to the road of success.

Grit, as a desirable characteristic, has been shown to enhance students’ engagement in schoolwork and reduce the likelihood of dropping out (M. Li, 2024; Slick & Lee, 2014). The negative relationship between grit and academic burnout has been consistently revealed among Chinese adolescents. For instance, Teuber et al. (2021) suggested that both aspects of grit (i.e., persistent effort and long-term interest) were negatively associated with exhaustion, a major component of burnout. Additional research has explained that by using grit to foster positive emotions and experience associated with school, one can reduce the likelihood of suffering from burnout (Özhan, 2021).

Although the moderating role of grit in academic stress and burnout remains underexplored, existing empirical studies have shown that grit can serve as a shielding factor to safeguard students from experiencing negative psychological outcomes, such as depression and thoughts of suicide (Kleiman et al., 2013; Zhang et al., 2021). Given its nature of being a psychological strength, grit can buffer the impact of adversity by promoting better mental functioning (Datu et al., 2021). Grit serves as a personal resource when integrated into the JD-R model, which fuels academic engagement and alleviates negative outcomes (Teuber et al., 2021). In the case of the current study, when facing excessive academic burdens, gritty students may find themselves less likely to experience academic burnout as grit can serve as a personal resource and protective factor (Tang et al., 2021). Such evidence allows us to adopt a similar perspective and hypothesize that grit may be a moderator in the relationship between academic stress and burnout.

### 2.4. The Present Study

**Figure 1:**
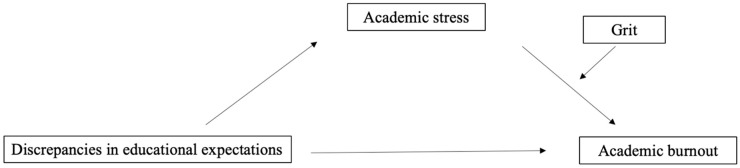
The hypothesized model.

This study adopts a quantitative cross-sectional design to empirically examine the mechanisms underlying discrepancies in educational expectations (both perceived by students and imposed by parents) and academic stress and burnout (Figure 1). According to Identity Control Theory (ICT), psychological distress can arise when individuals perceive a mismatch between their self-defining standards (e.g., personal educational expectations) and feedback from others (e.g., perceived parental or teacher expectations), disrupting the process of identity verification (Burke, 2007). Grounded in previous research and the Job Demands–Resources (JD-R) model, these discrepancies can be conceptualized as an external demand, which may contribute to increased stress and, ultimately, academic burnout. Meanwhile, grit functions as a personal resource that may help mitigate the negative effects of stress on burnout. By integrating ICT with the JD-R model, this study explores a moderated mediation model on how discrepancies in educational expectations influence students’ academic well-being. Obtaining a better understanding of the mechanistic interactions between these constructs will provide more insights into how to alleviate stress and burnout in the learning process, especially from the perspectives of family and parents.

## 3. Method

### 3.1. Participants

Participants were recruited from a public middle school in Zhengzhou, Henan Province. All ninth graders from this school were invited to participate, and 581 responded. After excluding 50 invalid responses, including missing data for main variables and answering trap questions incorrectly, a total of 531 valid questionnaires were included as the final sample. The average age of the participants was 14.77 years old, and 250 (47.08%) were girls.

### 3.2. Procedures

This study received approval from the ethics committee at the first author’s university. A cross-sectional survey design was employed. Data collection took place over a one-week period in March 2023. The questionnaire was distributed online through the platform “Questionnaire Star” to the WeChat groups of every class, with the support and permission of school administrators. The inclusion criteria specified that participants must be currently enrolled in the ninth grade at this middle school, aged between 13 and 16. Students were excluded if consent was not obtained or if their responses were incomplete. The questionnaire included four key measurements (i.e., educational expectations, academic stress, academic burnout, and grit), along with basic demographic and family SES information (i.e., gender, age, parents’ educational level, and parents’ income level). Informed consent was obtained online from both students and their parents prior to participation.

### 3.3. Measures

#### 3.3.1. Discrepancies in Educational Expectations

Two questions adopted from Y. Wang and Benner’s (2014) study were used to examine the discrepancies in educational expectations. Students’ educational expectations were assessed by the following question: “How far in school do you think you will go?” Another question assessing their perceptions of parental expectations was, “How far in school do you think your parents want you to go?” Both questions utilized a 5-point rating scale ranging from 1 to 5, where 1 = finish middle school, 2 = finish high school, 3 = obtain a bachelor’s degree, 4 = obtain a master’s degree, and 5 = attain beyond a master’s degree. Discrepancies were calculated by subtracting students’ expectations from parental expectations. A larger absolute value indicated greater discrepancies. The set of questions has been validated and confirmed to be reliable in previous studies (e.g., Man et al., 2021). Cronbach’s α coefficient in this study was 0.78.

#### 3.3.2. Academic Stress

Perceived academic stress was assessed using one of the local scales specifically tailored for the Chinese context (Liu & Lu, 2012). The scale consisted of seven items (e.g., “To solve the problems assigned by teachers is so difficult”), based on a 4-point Likert-type scale, where 1 represented “strongly disagree” and 4 represented “strongly agree”. Higher scores indicated higher academic stress levels. The scale was developed in the Chinese context and was found to be valid with a decent Cronbach alpha (e.g., J. L. Wang et al., 2020). In this study, Cronbach’s α coefficient was 0.89.

#### 3.3.3. Academic Burnout

Academic burnout was assessed using the Chinese adaptation of the Maslach Burnout Inventory-Student Survey (MBI-SS; Schaufeli et al., 2002), which has been validated in the Chinese context (Hu & Schaufeli, 2009). Based on the three subscales, the MBI-SS consisted of 15 items, each rated on a 7-point Likert scale ranging from 0 (never) to 6 (always) that separately measured exhaustion (e.g., “I feel emotionally drained by my studies”), cynicism (e.g., “I doubt the significance of my studies”), and inefficacy (reverse-coded; e.g., “I can effectively solve the problems that arise in my studies”). Higher scores suggested higher levels of academic burnout. Cronbach’s α coefficient was 0.93 in the current study.

#### 3.3.4. Grit

The Chinese validated adaptation of the Short Grit Scale (Duckworth & Quinn, 2009; J. Li et al., 2018) was applied to evaluate grit levels. This comprehensive scale contains eight items measuring grit using two subscales: perseverance of effort (e.g., “I finish whatever I begin”) and consistency of interest (e.g., “I often set a goal but later choose to pursue a different one”). All items were rated using a 5-point Likert-type scale ranging from 1 (not at all like me) to 5 (very much like me). Cronbach’s α coefficient was 0.81.

### 3.4. Statistical Analyses

All data analyses were conducted by using IBM SPSS, version 26. First, the common method bias was examined, ensuring that no single factor accounted for the majority of the variance. Descriptive statistics and Pearson correlation analyses were then performed to explore the distributions and interrelationships among the main study variables. Next, PROCESS macro Model 4 (Hayes, 2012) was used to test academic stress as the mediator, and the moderated mediation effect was examined by Model 14 of the PROCESS macro.

## 4. Results

### 4.1. Common Method Bias

Common method bias refers to the problem that arises when the same respondent provides the predictor and criterion variable in self-report questionnaires (Podsakoff et al., 2003). Thus, Harman’s single-factor test (Podsakoff & Organ, 1986) was utilized to uncover any potential bias. The results indicated that seven factors had eigenvalues greater than 1, and the first factor accounted for 38.15% of the total variation, which is below the critical standard of 40%. Hence, the effect of common method bias was insignificant in the current study.

### 4.2. Preliminary Analyses

Table 1 shows the means, standard deviations, and correlations among all variables. As we expected, academic burnout was positively related to expectation discrepancies (*r* = 0.35, *p* < 0.01) and academic stress (*r* = 0.79, *p* < 0.01). Additionally, grit was negatively related to expectation discrepancies (*r* = −0.27, *p* < 0.01), academic stress (*r* = −0.67, *p* < 0.01), and academic burnout (*r* = −0.75, *p* < 0.01). As parents’ income was not significantly related to any of the main variables, further analyses did not control for it as one of the covariates.

Table 2 presents the distribution of expectation discrepancies. As mentioned earlier, the value of discrepancies was represented using parental educational expectations minus children’s own. Specifically, most students (*n* = 331) perceived themselves and their parents as having equal expectations. This was followed by 161 students who perceived their parents’ expectations as higher than their own. Lastly, only 39 students thought their expectations were higher than their perceived parental expectations.

### 4.3. Testing of the Mediation Model

The role of academic stress as a mediator in the relationship between expectation discrepancies and academic burnout was examined using Model 4 of the PROCESS macro (Hayes, 2012). After controlling for participants’ gender, age, and parents’ educational level as covariates, the results (see Model 1 in Table 3) showed that such discrepancies were positively related to academic burnout (*β* = 0.33, *p* < 0.001). Furthermore, the discrepancies were also positively related to academic stress (*β* = 0.28, *p* < 0.001) (see Model 2). After including academic stress as a mediator in the model, the discrepancies were still significantly related to academic burnout (*β* = 0.13, *p* < 0.001). Meanwhile, the impact of academic stress on academic burnout was also significant (*β* = 0.73, *p* < 0.001), which indicated a partial mediation (see Model 3). These results demonstrated that the discrepancies in educational expectations were linked with burnout both directly and indirectly, with academic stress serving as a mediator.

### 4.4. Testing of the Moderated Mediation Model

**Figure 2:**
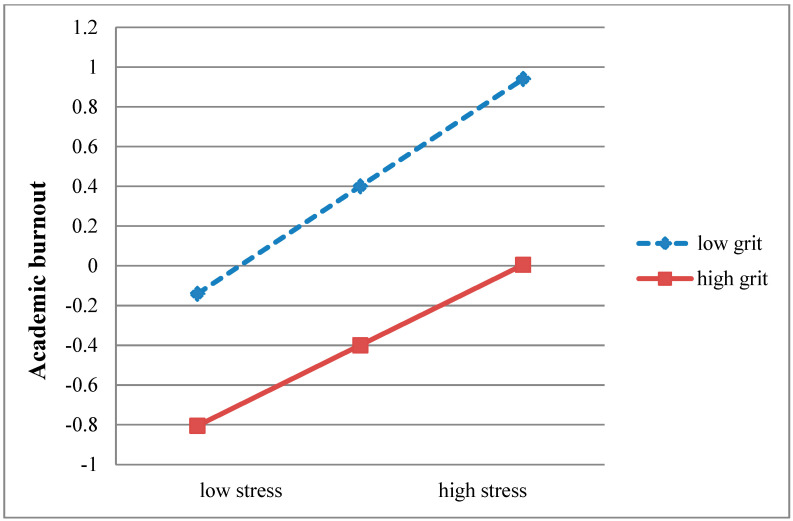
Interactive effect of academic stress and grit on academic burnout.

PROCESS macro Model 14 (Hayes, 2012) was used to test whether grit moderated the relationship between academic stress and academic burnout after accounting for gender, age, and parents’ educational level as covariates. As shown in Table 4, the interaction between academic stress and grit negatively related to academic burnout (*β* = −0.07, *p* < 0.001), demonstrating that grit moderated the effect of academic stress on academic burnout. To clearly display the moderating role of grit, the effect of academic stress on burnout was plotted at ±1 SD levels of grit (see Figure 2). The results suggested that, compared with students with higher levels of grit (*β* = 0.41, *p* < 0.001), those with lower levels (*β* = 0.54, *p* < 0.001) were more likely to experience academic burnout when facing stress in their studies.

## 5. Discussion

This study tested the relationship between discrepancies in students’ educational expectations and their perceived parental expectations and academic burnout, focusing on the mediator of academic stress and the moderator of grit. The findings showed that the discrepancies were related to academic burnout directly and indirectly through the mediation of academic stress. In addition, the findings supported that grit moderated the relation between academic stress and burnout.

Over 30% of the participants perceived their parents’ expectations as being higher than their own, whereas less than 10% thought that their parents’ expectations were lower. Similar results were reported in the study by Y. Wang and Benner (2014), in which almost twice as many students perceived their parents’ educational expectations as exceeding their own, compared to those perceiving downward discrepancies. One possible explanation is that in Confucian cultural settings, Chinese parents place the utmost value on children’s education by carrying high expectations for educational and career attainment, hoping for more effort and better grades in school (Ng & Wei, 2020). Research has shown that Chinese mothers, for example, expressed higher expectations for their children’s academic achievement than American mothers, even as early as first grade (Ng et al., 2017). Students could perceive such an emphasis on education, especially through the prevalent authoritarian parenting style in China (Porter et al., 2005).

Perceived higher expectations led to greater discrepancies, which were associated with more academic stress, as indicated by the first half of the mediation model in this study. The discrepancies between parental educational expectations and students’ own highlight the difficulty of living up to expectations from family, becoming a source of stress (L. F. Wang & Heppner, 2002). Costigan et al. (2010) suggested that such stress in fulfilling expectations may come at the expense of students’ overall well-being. The second half of the mediating model revealed that academic stress is positively related to academic burnout among Chinese middle school students. This result aligns with findings from previous research that adolescents in China bearing high levels of academic stress were more likely to experience burnout, consequently leading to lower physical and mental well-being, such as depression (Jiang et al., 2021) and poor sleep quality (Yan et al., 2018). Middle school and high school students in China are under a significant amount of stress from the Gaokao, a high-stakes exam for higher education selection that is notorious for the overwhelming pressure it places on students (Muthanna & Sang, 2016). Long-term academic stress was found to be one of the main causes of academic burnout, resulting in emotional exhaustion, cynical attitudes, and reduced self-confidence (Jung et al., 2015).

The expectation discrepancies influenced academic burnout through the mediator of academic stress. This is a logical conclusion congruent with the JD-R model, as the discrepancies could be one of the study demands that cause academic stress, which successively leads to burnout in the health impairment path. Thus, the greater the discrepancies between parents and students in terms of educational expectations, the more stress students have to deal with, which in turn is more likely to trigger burnout syndrome.

Beyond the mediating role of academic stress, the results indicated that expectation discrepancies directly impacted academic burnout. This finding indicates that the difficulty of bridging the expectations gap has a detrimental effect on students’ academic health, especially when the discrepancies are significant. The effect of discrepancies can be explained by prior research showing that, in contrast to White American students, Asian American students were more inclined to worry about not living up to their parents’ academic expectations (Saw et al., 2013). Burnout may arise if students are chronically concerned about not fulfilling parental expectations.

Moreover, the current study found that grit functioned as a moderator in the second half of the moderated mediation model. In particular, those with higher levels of grit were less prone to experiencing academic burnout than those with lower levels. According to the transactional model of stress and coping (TSC), stress arises from the dynamic interaction between a person and their environment (Lazarus & Folkman, 1984). When facing a stressful situation, people appraise whether their relevant coping resources are sufficient to deal with the situation. Studies show that resilience-related approaches, such as grit, mindset, and hardiness, are excellent resources for coping with adversity (Frydenberg & Frydenberg, 2017). The findings of this study add evidence to the resilience trait of grit, as adolescents whose grit levels were higher were found to be less vulnerable to burnout when managing learning-related stress. These results align with the previous research on the JD-R model, which suggests that personal resources, such as grit, enhance engagement and protect against burnout (Corso-de-Zúñiga et al., 2020). Taken together, these findings suggest that academic burnout is influenced by both external demands (e.g., discrepancies in parental expectations) and personal resources (e.g., grit). The JD-R model provides a robust framework for understanding how misaligned expectations function as stressors that impair well-being, while personal resilience factors help mitigate the negative effects of stress on burnout.

### 5.1. Limitations and Implications

This study has several limitations. First, although the cross-sectional data produced useful results, they cannot determine causal relationships between the variables. Future studies could utilize an experimental or longitudinal design to verify the causal relationships between the discrepancies in expectations, academic stress, and academic burnout. Second, all the data were gathered through a self-report survey, which could have resulted in measurement bias. While this approach is consistent with Identity Control Theory (ICT)—which emphasizes how individuals respond to perceived feedback—this unidirectional measure does not account for parents’ actual expectations. Relying only on students’ perceptions may result in measurement bias, as these perceptions might not accurately reflect the expectations held by parents. Future research could include data from different sources of informants (e.g., parents) to better examine the validity of the current results. Third, no generalized conclusions could be reached since the findings were only derived from one middle school in China. More research is required across different age groups and contexts to test the consistency of the current results. Fourth, this study examined only the construct of grit as the moderator. Other resilience-related factors, such as hardiness, mindset, and goal orientation, could be included in further research to test their possible moderating effects. Last, in addition to the expectation discrepancies between parents and students, other studies could focus on the discrepancies between teachers and students to examine whether this is also a potential stress-related factor.

Despite the limitations, the current findings provide evidence for the existing research on the relationship between expectation discrepancies and academic burnout, along with the mediator of academic stress and the moderator of grit. Theoretically, this model has not been previously explored, and the current study provides insights into how discrepancies in educational expectations between students and their parents could negatively affect students’ academic health. Several practical implications can be identified for alleviating academic burnout among middle school students. First, the discrepancies in expectations between parents and children could contribute to academic burnout directly. To reduce such discrepancies, parents are encouraged to communicate regularly with their children and stay informed about their actual academic performance. This approach can help prevent unrealistic expectations and, when necessary, support the adjustment of parental expectations. Second, regarding the mediating role of perceived stress, more workshops can be hosted in schools to introduce strategies to cope with stress within school settings (Houston et al., 2017). Previous research has demonstrated the malleability of grit and that successful interventions can effectively enhance grit among adolescents (Alan et al., 2019). Therefore, programs designed to develop grit can be implemented in schools to better equip students with the psychological capital needed to protect themselves against academic burnout.

### 5.2. Conclusions

In conclusion, the present study demonstrated that discrepancies between adolescents’ own educational expectations and their perceived parental expectations were positively associated with academic burnout, with academic stress serving as a mediating factor. This highlights the psychological burden students may experience when their expectations are misaligned with those of others. Additionally, grit was found to moderate the relationship between academic stress and burnout, suggesting that students with higher levels of grit may be more resilient to the negative emotional effects of academic stress. These findings underscore the importance of both reducing expectation discrepancies and fostering internal resources such as grit. Interventions aimed at improving parent–child communication, aligning academic goals, and strengthening students’ perseverance may help mitigate academic stress and burnout, ultimately supporting both academic success and emotional well-being.

## Figures and Tables

**Table 1:** Means, SDs, and correlations among variables (N = 531).

	*M*	*SD*	1	2	3	4	5	6	7	8
Expectation discrepancies	0.31	0.78	-							
2. Academic stress	17.4	4.38	0.29 **	-						
3. Academic burnout	50.73	20.7	0.35 **	0.79 **	-					
4. Grit	25.03	5.77	−0.27 **	−0.67 **	−0.75 **	-				
5. Gender	0.47	0.50	0.04	0.18 **	0.18 **	−0.22 **	-			
6. Age	14.77	0.63	0.05	−0.10 *	−0.08	0.12 **	−0.18 **	-		
7. Parents’ income	3.53	1.43	−0.07	−0.04	0.01	0.02	−0.09 *	0.001	-	
8. Parents’ education	2.76	0.87	−0.06	−0.13 **	−0.13 **	0.08	−0.01	−0.17	0.25 **	-

Note. * *p* < 0.05, ** *p* < 0.01.

**Table 2:** Frequencies of expectation discrepancies (N = 531).

	*n*	%
Child > Parent	39	7.3
Child = Parent	331	62.3
Child < Parent	161	30.4

**Table 3:** The mediating model of academic stress (N = 531).

Variables	Model 1 (Academic Burnout)		Model 2 (Academic Stress)		Model 3 (Academic Burnout)	
	*β*	*t*	*β*	*t*	*β*	*t*
Gender	0.30	3.86 ***	0.30	3.78 ***	0.08	1.51
Age	−0.11	−1.72	−0.13	−2.07 *	−0.01	−0.24
Parents’ education	−0.12	−2.67 **	−0.12	−2.61 **	−0.03	−1.06
Expectation discrepancies	0.33	8.39 ***	0.28	6.78 ***	0.13	4.78 ***
Academic stress					0.73	26.38 ***
*R* ^2^	0.16		0.13		0.64	
*F*	25.85 ***		19.62 ***		187.24 ***	

Note. *** *p* < 0.001, ** *p* < 0.01, * *p* < 0.05.

**Table 4:** The moderated mediating effect of expectation discrepancies on academic burnout (N = 531).

Variables	Academic Stress		Academic Burnout	
	*β*	*t*	*β*	*t*
Gender	0.30	3.78 ***	0.01	0.29
Age	−0.13	−2.07 *	0.02	0.58
Parents’ education	−0.12	−2.61 **	−0.04	−1.55
Expectation discrepancies	0.28	6.78 ***	0.09	3.89 ***
Academic stress			0.47	15.26 ***
Grit			−0.40	−12.62 ***
Academic stress × grit			−0.07	−3.52 ***
*R* ^2^	0.13		0.73	
*F*	19.62 ***		199.59 ***	

Note. *** *p* < 0.001, ** *p* < 0.01, * *p* < 0.05.

## Data Availability

The data that support the findings of this study are available from the corresponding author upon reasonable request.
